# Multigenerational metabolic disruption: Developmental origins and mechanisms of propagation across generations

**DOI:** 10.3389/ftox.2022.902201

**Published:** 2022-08-19

**Authors:** Daniel D. Davis, Carlos Diaz-Castillo, Raquel Chamorro-Garcia

**Affiliations:** Department of Microbiology and Environmental Toxicology, University of California, Santa Cruz, Santa Cruz, CA, United States

**Keywords:** DOHAD, metabolic disruption, transgenerational inheritance, exposome, endocrine-disrupting chemicals

## Abstract

It has been long known that the environment plays a critical role in the etiology of disease. However, it is still unclear how the large variety of environmental factors humans are exposed to interact with each other to lead to disease. Metabolic disorders are just one example of human disorders that have been associated with environmental exposures. Obesity and type 2 diabetes have become a health and economic burden worldwide as the number of affected people has tripled in the last 40 years. Animal and human studies have shown a strong association between exposure to environmental chemicals during critical windows of susceptibility such as periconception, prenatal, and early life, whose effect can persist through development and across generations. However, little is known about the mechanisms driving this persistence. Here, we review historical and current knowledge on the effect of exposure to environmental factors during *in utero* development and discuss mechanisms for these disorders to be propagated across generations.

## Introduction

Metabolic disorders, such as obesity and type 2 diabetes are examples of diseases that can have multifactorial origins. Lifestyle choices, such as hypercaloric diets and sedentarism, have been strongly associated with the increase in metabolic disorders in the last 40 years. For example, between 1999 and 2017, obesity prevalence increased from 30.5% to 42.4% in the United States (Hales et al., 2020). In Europe, estimates in 2008 showed ∼53% of the population is overweight, with 36% considered pre-obese and 17% obese (Legler et al., 2015), indicating that increasing trends in obesity are a worldwide health concern (Hales et al., 2017). In parallel, cases of type 2 diabetes are estimated to increase from 464 million people in 2019 to 700 million by 2045 worldwide, with developing countries leading this trend (Menke et al., 2015). Taken together, obesity and obesity-related diseases are contributing to increasing trends in preventable premature death (CDC 2022) (Hales et al., 2020).

Beyond the toll metabolic disease has on the health of individuals, global rates of such diseases have a key economic burden worldwide. The cost of treating overweight and obese individuals in the United States is 9.9% and 42.7% higher, respectively, than the healthcare costs of someone with a healthy weight (Tsai et al., 2011). The economic impact of obesity is not just limited to direct medical care costs, as obesity can also impact workplace productivity (Hamilton et al., 2018). The total direct and indirect healthcare costs of obesity are estimated at ∼ $200 billion annually (Biener et al., 2017). In order to change the current trends of metabolic diseases, it is necessary to have a deep understanding of the contributing factors as well as the interventions that can be implemented to prevent these diseases.

Considering the global implications of both obesity and type-2 diabetes it is necessary to determine the factors that are contributing to increasing the prevalence of these conditions. They both have been traditionally associated with high calorie intake and sedentary lifestyle. There is also evidence showing that exposure to certain environmental factors, such as endocrine-disrupting chemicals, can play an important role in contributing to these phenotypes (Merrill et al., 2019) that on many occasions has been shown to propagate across generations in animal models. However, there is no consensus about the epigenetic mechanisms of action of these environmental factors to lead to multigenerational disease and there is little information about how these alterations are propagated across generations. Here, we review current knowledge on the field of transgenerational epigenetic inheritance, describe a new mechanism of action that involves alterations of nuclear genome organization and propose a new model of propagation of these alterations across generations.

## Endocrine-disrupting chemicals and environmental determinants of disease

The exposome refers to all the environmental factors, including physical and chemical factors, social aspects, and lifestyle choices, humans are exposed to during our lifespan, playing a critical role in human health, and disease (Wild 2005; Vermeulen et al., 2020). In recent years, it has been shown that exposure to certain chemicals present in the environment can contribute to increasing susceptibility to metabolic disruption (Heindel 2019). Disparities found in the amount of chemicals individuals are exposed to depending on characteristics such as demographics, and socioeconomic status, are bringing attention to environmental determinants of disease that should be studied under the prism of environmental justice (Johnston and Cushing 2020). For example, an association between higher levels of chemicals, lower household income, and lower education has been previously observed, and people classified as living in poverty had higher exposure to many harmful chemicals, including lead, pesticides, phthalates, and polycyclic aromatic hydrocarbons (Johnston and Cushing 2020). Given the large variety of factors encompassed by the exposome that can contribute to disease, considering single factors to explain complex diseases that are influenced by the environment limits our understanding of the origins of these diseases.

Endocrine-disrupting chemicals (EDCs) are a subset of chemical factors found in the environment that alter the function of the endocrine system, potentially leading to a variety of adverse health outcomes that include metabolic disorders, neurological disorders, infertility, and endocrine cancers (Anway et al., 2005; Cohn et al., 2007; Carwile and Michels 2011; Legler et al., 2015; Liu et al., 2018; Merrill et al., 2019). The endocrine system comprises glands that produce hormones throughout the body that are released into the bloodstream or fluid surrounding the cells. Hormones are recognized by receptors in surrounding organs and tissues where they elicit a response. Biological processes regulated by the endocrine system include blood sugar control, body growth, and energy production, or the differentiation, growth, and function of reproductive organs. Hormones are found in the body at very low levels, and their production is tightly regulated at any given time depending on body needs (Gore et al., 2015). Alterations in the timing of release and/or amount of hormones can lead to disease. Since hormones are present in very low amounts in the body, external chemicals that disrupt hormone functions, such as EDCs, are not needed in very high amounts to disturbing body functions.

EDCs exposure during *in utero* development can lead to an increased risk of developing diseases in adulthood such as obesity and type-2 diabetes (Braun 2017). A study performed primarily on a cohort of San Francisco low-income female Latinas found that fetuses experienced higher exposure to environmental pollutants than their mothers, probably due to bioaccumulation and lower clearance of the chemicals (Morello-Frosch et al., 2016). Considering that developing embryos are significantly smaller than individuals at any other stage in life, these data suggest that the level of exposure to environmental factors is significantly higher during *in utero* development than at any other time throughout a lifetime, which can lead to adverse health outcomes later in life (Woodruff et al., 2011). In addition to the effects of EDC exposure during *in utero* development has in metabolic disruption later in life, childhood exposure can also have health-related consequences during adulthood, including high blood pressure, high glucose plasma levels, and a high prevalence of dyslipidemia which will skew the current metabolic trends towards an increasing percentage of overweight and obese populations (Boyd et al., 2005; Ogden et al., 2016).

## Developmental origins of health and disease

The Developmental Origins of Health and Disease (DOHaD) hypothesis poses that exposure to certain environmental factors during the preconception, prenatal, and/or early postnatal periods can increase disease susceptibility later in life (Barker 2007).

Observations made in the 1930s led William Ogilvy Kermack to argue that adult disease could largely be explained by the environment the individual was exposed to during childhood and puberty (Smith and Kuh 2001). In the 1940s, the events that took place at the end of World War II in the Netherlands, where the population was severely impacted by a famine period of ∼8 months (i.e., the Dutch Famine), supported the hypothesis that environmental exposures during pregnancy would affect the health of the developing children later in life (Rooij et al., 2021). That children whose mothers were exposed to the famine during preconception, or the first trimester of pregnancy, had lower quality of life and increased susceptibility to disease compared to children whose mothers were exposed at other windows during pregnancy drew attention to early stages of development or even before fertilization, as critical for susceptibility to disease later in life (Rooij et al., 2021). Although throughout the 20th Century there was supporting evidence showing that the environment to which mothers are exposed during preconception, pregnancy, and shortly after birth played a role in later disease outcomes in the offspring, it was not until the work done by David Barker between the 1960s–1980s when that hypothesis started gaining momentum. Barker (1966) made the initial observation that the intra-uterine environment played a critical role in children’s development (Barker 1966). Later observations showed a strong association between geographical location (i.e., environment) and infant mortality (Barker and Osmond 1986). Most of the adverse health outcomes initially observed in these studies were associated with metabolic disruption, including obesity, type 2 diabetes, and metabolic syndrome (Rebecca et al., 2005; Roseboom et al., 2006). Later analyses identified other diseases such as cognition deficiencies, infertility, or greater predisposition to certain types of cancer (Rooij et al., 2021). Although many of these diseases have been traditionally explained using genetics, the publication of the human genome alongside the development of high throughput methodologies revealed that genetic factors would only explain a very small portion of the cases and placed the focus on “other” heritable factors driving these phenotypes. For example, in the case of obesity, <3% of obesity cases can be explained due to genetic alterations (Locke et al., 2015), which suggested that mechanisms other than genetics are involved in disease.

## Basic research approaches to understanding human disease

Seminal work published in the 2000s using rodent models supported the hypothesis that non-genetic mechanisms of action, mostly focused on perturbations of DNA methylation, might be involved in disease development upon exposure to environmental factors (Weaver et al., 2004; Anway et al., 2005). Michael Meaney and collaborators showed that maternal licking and grooming behavior to their pups perturbed DNA methylation around the gene encoding the glucocorticoid receptor in the hippocampus that persisted throughout adulthood (Weaver et al., 2004). This example of non-genetic programming of maternal behavior could be reversed by cross-fostering the progeny shortly after birth, suggesting that maternal care directly influences the methylation status of the promoter region of the glucocorticoid receptor gene (Weaver et al., 2004). Michael Skinner and collaborators demonstrated for the first time that exposure to the EDC vinclozolin altered sperm quality and DNA methylation in multiple generations (Anway et al., 2005). These studies opened a new paradigm in our understanding of environmental determinants of disease, the role of the environment in multigenerational disease, and the mechanisms of transgenerational non-genetic inheritance.

After the observations made in animal models, human data analyzing DNA methylation perturbations associated with environmental exposures were published, showing that those mechanisms of action may also apply to human populations. Work performed by Keith M. Godfrey and collaborators showed that low maternal carbohydrate intake in the early stages of pregnancy led to increased adiposity in their children associated with hypermethylation of the retinoid X receptor alpha (RXRα) promoter region (Godfrey et al., 2011). RXRα is the heterodimeric partner of the peroxisome proliferator-associated receptor gamma (PPARγ) and, together, they regulate fat development, suggesting that functional alterations of these factors can contribute to increasing susceptibility to metabolic disease. Analyses performed in the Dutch Famine cohort show the effects of environmental exposures (i.e., dietary restriction) during different stages of pregnancy. The phenotypes observed in the offspring are different but somewhat associated with metabolic disorders, including altered glucose metabolism and a higher incidence of cardiovascular disease and obesity. Analyses performed in this cohort showed a perturbation of DNA methylation associated with peri-conceptional exposure to the famine (Heijmans et al., 2008), suggesting that early-life experiences can alter the epigenome and that those alterations persist throughout life.

Taken together, animal, and human studies demonstrated that exposure to environmental perturbations during gestation, particularly during early gestation, plays a critical role in the health of the offspring later in life by introducing non-genetic perturbations in their genomes that can potentially propagate to future unexposed generations.

## Transgenerational epigenetic inheritance

Transgenerational epigenetic inheritance (TEI) refers to those non-genetic perturbations that have the potential of being propagated across generations, having important implications for multigenerational disease and evolution (Miska and Ferguson-Smith 2016). Traditional views of mechanisms of propagation across generations question the possibility of propagation of epigenetic traits as they are reprogrammed during embryogenesis (Heard, 2014). As such, the chances for epigenetic perturbations to be inherited are reduced.

Michael Skinner and collaborators pioneered the field with their work on exposure to vinclozolin during epigenetic reprogramming and transgenerational disease (Anway et al., 2005). The first observations about potential mechanisms of action focused on alterations in DNA methylation patterns in the sperm across multiple generations (Anway et al., 2005). Those findings, however, did not explain how perturbations of DNA methylation were passed from one generation to the next, considering the erasure of DNA methylation during embryogenesis in each generation. Similar limitations were found when looking at other epigenetic marks, such as histone modifications, which are also reprogrammed during embryogenesis (Heard, 2014). Altered presence of non-coding RNAs upon fertilization has also been associated with disease later in life (Carone et al., 2010). However, most paternal RNAs are degraded shortly after fertilization, and although they might have already led to perturbations in the zygote before degradation, their propagation to future generations needs to be demonstrated (Heard, 2014). More recently, nuclear genome organization, which refers to the three-dimensional (3D) spatial organization of the genome within the nucleus has been considered as a potential epigenetic mechanisms of gene expression regulation that can be propagated across generations. The 3D spatial organization is involved in many different gene regulatory processes by regulating accessibility of transcription factors to certain regions and creating topologically associated domains (TADs) that will bring together regions of the genome that would be far apart if the genome followed a linear organization (Misteli 2020).

Our research suggests that exposure to the EDC tributyltin (TBT) at critical windows of susceptibility can contribute to perturbations of nuclear genome organization that subsequently will alter other genomic traits such as DNA methylation and gene expression (Chamorro-Garcia et al., 2017; Diaz-Castillo et al., 2019). We showed that *in utero* exposure to TBT leads to perturbations of the expression of the genome that are consistent with perturbations of nuclear genome organization (Chamorro-Garcia et al., 2017; Diaz-Castillo et al., 2019). Genes associated with chromatin organization were differentially expressed in animals ancestrally exposed to TBT compared to control animals (Chamorro-Garcia et al., 2017; Diaz-Castillo et al., 2019). We found that approximately 700 genes from the gene ontology term GO:0006325 (Chromatin Organization) were differentially expressed in liver, gonadal adipose tissue, and mesenchymal stem cells (fat precursor) with 668 genes overlapping between the three tissues. These genes represented processes such as modification of histones (histone acetyl and deacetyl transferases, and histone demethylases), chromosome stability, and nucleosome assembly. These findings led us to hypothesize that TBT alters the expression of genes involved in the establishment of chromatin organization, which would subsequently influence chromatin organization through mitosis and meiosis and, as such, can be propagated across generations (Chamorro-Garcia et al., 2017; Diaz-Castillo et al., 2019).

There have been other EDCs that lead to multigenerational metabolic disruption including, bisphenol A, methoxychlor, phthalates, jet fuel, dichlorodiphenyltrichloroethane (DDT), benzo[a]pyrene, and glyphosate (Manikkam et al., 2012, 2013, 2014; Skinner et al., 2013; Tracey et al., 2013; Kubsad et al., 2019). In these studies, the F1 and F2 generations were exposed to the chemicals as the treatment was performed during F0 pregnancy. Alterations of DNA methylation in sperm were observed upon exposure to those chemicals. Alterations in sperm histone retention and non-coding RNAs were also observed in animals directly or ancestrally exposed to DDT. Interestingly, integrative analyses of DNA methylation, histone retention and non-coding RNAs alterations in the sperm showed an overlap in the presence of this events (Beck et al., 2021).These findings show a regionalization in the location of epigenetic marks which might be consistent with alterations of nuclear genome organization that will condition their location in the genome.

This new perspective for TEI bypasses the limitations stemming from the epigenetic reprogramming events during development (Heard, 2014). In our model, alterations of chromatin organization occur prior to epigenetic reprogramming during development, poising the genome to differentially expressed genes involved in chromatin organization (Diaz-Castillo et al., 2019). In this model, the amount and nature of proteins involved in chromatin organization (e.g., histones, histone modifiers, proteins involved in chromosome stability and nucleosome formation) would be altered in the cytoplasm. Upon cell division, these proteins are deposited in the daughter cells with an altered ratio compared to the control group, which will subsequently affect the establishment of nuclear genome organization in the new cells. This mechanism will likely alter the chromatin accessibility in certain regions to other epigenetic modifiers such as DNA methyltransferases and histone modifiers. These perturbations are maintained across cell divisions in somatic tissues and meiosis in the germline that will lead to future generations. Since the number of elements involved in chromatin organization will vary from individual to individual due to natural variation, it is expected that chromatin establishment after each division will be slightly different in each individual. As such, rather than expecting a replicative epigenetic mechanism of propagation (e.g., faithful replication of alterations in epigenetic marks), our observations suggest a process of reconstruction of the alterations that will be slightly different in every individual and generation (Diaz-Castillo et al., 2019). Since alterations in chromatin structure will slightly vary, the transcription machinery will have variable accessibility to genes involved in metabolic process, leading to phenotypes that may vary among individuals. Instead of looking at a limited number of genes that can explain the phenotype associated to any one exposure, our results propose to take a holistic approach and look into global gene expression alterations associated to pathways linked to specific locations in the genome. Whether these mechanisms apply to the human population is currently unknown as there is very limited information coming from human cohorts.

## Conclusion and future directions

That environmental factors play an important role in the onset of multigenerational metabolic conditions has been demonstrated in human studies and in animal models. Despite much effort in trying to elucidate the mechanisms through which environmental factors lead to transgenerational metabolic disorders, there is currently no consensus as to how this occur and how those epigenetic mechanisms scape the process of epigenetic reprogramming during embryogenesis each generation. The model that we are proposing bypasses those limitations and embraces previously described mechanisms of transgenerational epigenetic inheritance such as DNA methylation, histone retention and differential expression of non-coding RNAs. Further integrative analyses are necessary to confirm these observations, which if confirm, would change our understanding of how environmental factors can contribute to multigenerational disease as well as open new paths for prediction and prevention of certain health conditions.
